# Polycystic ovary syndrome and mitochondrial dysfunction

**DOI:** 10.1186/s12958-019-0509-4

**Published:** 2019-08-16

**Authors:** Jingshun Zhang, Yigang Bao, Xu Zhou, Lianwen Zheng

**Affiliations:** 1 Reproductive Medical Center, Department of Obstetrics and Gynecology, The Second Hospital of Jilin University, Changchun, Jilin China; 2 College of Animal Sciences, Jilin University, Changchun, Jilin China

**Keywords:** Polycystic ovary syndrome, Oxidative stress, Hyperandrogenism, Insulin resistance, Obesity, Abnormal follicular development, And inflammation

## Abstract

Polycystic ovary syndrome (PCOS) is a prevalent hormonal disorder of premenopausal women worldwide and is characterized by reproductive, endocrine, and metabolic abnormalities. The clinical manifestations of PCOS include oligomenorrhea or amenorrhea, hyperandrogenism, ovarian polycystic changes, and infertility. Women with PCOS are at an increased risk of suffering from type 2 diabetes; me\tabolic syndrome; cardiovascular events, such as hypertension, dyslipidemia; gynecological diseases, including infertility, endometrial dysplasia, endometrial cancer, and ovarian malignant tumors; pregnancy complications, such as premature birth, low birthweight, and eclampsia; and emotional and mental disorders in the future. Although numerous studies have focused on PCOS, the underlying pathophysiological mechanisms of this disease remain unclear. Mitochondria play a key role in energy production, and mitochondrial dysfunction at the cellular level can affect systemic metabolic balance. The recent wide acceptance of functional mitochondrial disorders as a correlated factor of numerous diseases has led to the presupposition that abnormal mitochondrial metabolic markers are associated with PCOS. Studies conducted in the past few years have confirmed that increased oxidative stress is associated with the progression and related complications of PCOS and have proven the relationship between other mitochondrial dysfunctions and PCOS. Thus, this review aims to summarize and discuss previous and recent findings concerning the relationship between mitochondrial dysfunction and PCOS.

## Background

Polycystic ovary syndrome (PCOS) is a lifelong illness, and its metabolic and reproductive syndromes manifest in all periods of life [1]. Its prevalence ranges from 8 to 13% in accordance with the investigated population and definitions used [2]. After several revisions, a consensus was reached that the diagnosis of PCOS should be based on the Rotterdam criteria, which include two of the three following characteristics: oligomenorrhea, hyperandrogenism (clinical or biochemical), and polycystic ovary on ultrasound after the exclusion of other endocrinopathies [3]. PCOS is frequently accompanied by abnormal follicular development, obesity, insulin resistance (IR), compensatory hyperinsulinemia, hyperandrogenism, and low-grade inflammation [4]. Ovarian hyperandrogenism, IR, hyperinsulinemia, and changes in follicular endocrine signals can interfere with follicular activation, survival, growth, and selection in women with PCOS. These effects result in the accumulation of small follicles around the ovary, polycystic morphology, and damage to follicular maturation and anovulation. The majority of women with PCOS are overweight, obese, or abdominally obese. Obesity can exacerbate the progression of various PCOS-related dysfunctions, such as anovulation, hyperandrogenism, IR, and inflammation. The progression of these dysfunctions subsequently increases adipogenesis and decreases lipolysis. Changes in fat–ovarian interactions, especially in the case of excess fat, exacerbate these events, which have adverse effects on follicular development and may damage oocytes [5, 6]. Obesity can aggravate IR and inflammation through the secretion of several inflammatory adipokines, sensitize thecal cells to luteinizing hormone (LH) stimulation, and upregulate ovarian androgen production. Thus, obese women with PCOS have more severe phenotypes, irregular menstruation, infertility, abortion, glucose intolerance, and metabolic syndrome than non-obese women with PCOS [7–9]. IR is a major factor of excessive adipogenesis in PCOS and is a key feature of the pathophysiology of PCOS. Generally, 75% of lean women and 95% of obese women with PCOS exhibit IR accompanied by severe metabolic disorders and late-stage complications [10]. IR and compensatory hyperinsulinemia can induce hyperandrogenemia by acting on the pituitary gland, ovaries, and liver [11]. An in vitro study has suggested that insulin can stimulate androgen secretion by theca cells, and androgen secretion is remarkably enhanced in theca cells from women with PCOS [12]. Similarly, insulin enhances adrenal androgen secretion in response to adrenocorticotropic hormone stimulation [13, 14], decreases the serum levels of sex hormone binding protein (SHBG), and then increases free androgen concentration. Thus, IR can promote the occurrence of hyperandrogenism from different aspects in patients with PCOS. Androgen excess is the main physiological and pathological mechanism of PCOS. It leads to reproductive, metabolic, and cosmetic changes that negatively affect the quality of life of patients with PCOS [15]. PCOS is caused by the vicious cycle of androgen excess. In this cycle, androgen excess impairs follicular development and promotes abdominal adipose tissue deposition by inducing IR and compensatory hyperinsulinism, which promotes androgen secretion by the ovaries and adrenal gland [16]. Recent studies have suggested that chronic inflammation may play a role in the pathogenesis of PCOS given that numerous inflammatory markers are elevated in women with PCOS. A relationship has also been found between proinflammatory status and PCOS, linked to polymorphism of gene coding for tumor necrosis factor-ɑ (TNF-ɑ), interleukin-6 (IL-6), and its receptor [17]. The inflammation associated with PCOS is explained in part by the coexistence of IR and obesity but is further fueled by androgen excess. PCOS is closely related to the dysfunction of adipose tissue. Similar to patients with PCOS, when exposed to androgen excess, adipocytes seem to be prone to hypertrophy. Hypertrophy of adipose tissue and androgen excess are related to IR. Inflammation is also closely associated with adipocyte hypertrophy, which leads to vascular compression, hypoxia, and elevated inflammatory markers [18]. Local inflammation in the ovary stimulates ovarian steroidogenic activity and theca cell proliferation and phosphorylation of the receptor, further increasing androgen excess and IR [19, 20]. The relationship between PCOS and inflammation may be confirmed by the association between the increased levels of inflammatory markers with the pathological development of other diseases, including type 2 diabetes(T2DM), atherosclerosis, and hypertension, in patients with PCOS [21–23]. In addition to IR, low-grade inflammation, obesity, and oxidative stress (OS) have been repeatedly shown to be prevalent in women with PCOS. Many studies have shown that the oxidative circulating markers in patients with PCOS are significantly higher than those in normal people; this condition is considered a potential cause of the pathogenesis of PCOS [24]. Mitochondria are important structures for regulating OS. Mitochondrial dysfunction has been proven to be a vital factor of T2DM, cardiovascular diseases, and cancer [25]. The role of mitochondrial gene and function disorders in the pathogenesis of PCOS has been investigated. It may account for several characteristics of PCOS, such as androgen excess, IR, obesity, abnormal follicular development, and inflammation [26]. Although the participation of mitochondrial dysfunction in the pathogenesis of PCOS is unclear, some results of current studies are important for the mechanism and treatment of PCOS. This review summarizes the recent findings for PCOS-associated mitochondrial genome (mtDNA) mutations and discusses the association among mitochondrial dysfunction and the clinical manifestations and progression of PCOS and related complications.

Mitochondria play a fundamental role in cell energy metabolism and apoptosis and are involved in signal transduction for cell proliferation [27]. Mitochondria translate nutrients into available energy and release reactive oxygen species (ROS) as by-products [28]. ROS are chemically reactive molecules containing oxygen. They include hydroxyl radicals (·OH), superoxide anions (O_2_·−), singlet oxygen (1O2), and hydrogen peroxide (H_2_O_2_) [29]. At appropriate levels, ROS have a pivotal impact on the biological functions of mammalian cells, such as multiplication, metabolism, gene expression, and immune response [30]. However, increased ROS production induces organism impairment. For example, the enhancement of OS in tissues and organisms causes related damage; harms mitochondrial components, such as mtDNA, proteins, and lipids; and induces mitochondrial-mediated apoptosis [31]. Typically, the deleterious effects of ROS are counteracted by a delicate antioxidant system that includes enzymatic antioxidants, such as superoxide dismutase (SOD), catalase (CAT), peroxidase (GPx), and paraoxonase, and nonenzymatic antioxidants, including glutathione (GSH), thioredoxin, thiols, vitamin C, vitamin A, vitamin E, selenium, and zinc [32]. Usually, SOD dismutate O_2_· − into H_2_O_2_. H_2_O_2_ is degraded to harmless water by CAT and GPx [33]. Thus, reductions in the amounts of these enzymes will result in excessive ROS production. GSH is a cofactor of several antioxidant enzymes. It can detoxify H_2_O_2_ and lipid peroxide by catalyzing GPx and regenerating vitamins C and E into their active forms [24]. Thiols are antioxidant compounds that neutralize oxidants into slightly toxic products [34]. Given that the direct measurement of OS in biological systems is not always feasible, the concentrations of various products of reactive oxygen metabolites are used to ascertain the redox state of tissues and mitochondrial function [35]. Malondialdehyde (MDA), total antioxidant capability (TAC), total antioxidant status (TAOS), mitochondrial membrane potential (MMP), advanced oxidation protein products (AOPPs), SOD, carbonyl, and GSH are common markers used to evaluate OS levels. MDA, a common marker of oxidant-mediated damage, is an end product of lipid peroxidation [36]. TAC refers to the capacity of scavenging harmful free radicals in blood and cells [37]. TAOS is described as the ability of serum to remove free radicals and protect cell structures from damage. AOPPs are novel biochemical markers of oxidant-mediated protein damage and represent a class of proinflammatory mediators [38]. Carbonyl can perform the oxidative modification of proteins and is an indicator of OS in plasma proteins [24]. MMP presents the ability to pump hydrogen ions through the inner membrane during oxidative phosphorylation (OXPHOS). The reduction in MMP is indicative of increased ROS production [39].

Mitochondria are controlled by the mitochondrial and nuclear genomes. MtDNA is a molecule with 16,569 base pairs (bps) and encode 22 tRNAs, two rRNAs, and 13 polypeptides that are indispensable for adenosine triphosphate (ATP) production [40]. All other proteins, including respiratory complex subunits, required for the normal function of mitochondria are encoded by nuclear genes [41]. The important enzyme involved in mtDNA replication and repair is DNA polymerase gamma, which is encoded by the POLG gene [42]. POLG is associated with numerous diseases. More than 150 different point mutations in POLG have been found to cause a wide range of diseases [43, 44]. Mitochondrial transcription factor A (TFAM), a major structural packaging protein of mtDNA, participates in mtDNA transcription and replication [45]. Defects in mitochondrial DNA replication result in mtDNA mutation, multiple deletions, and mtDNA molecule depletion [46]. Moreover, mtDNA has a higher mutation rate than nuclear DNA because it lacks histone protection and a DNA damage repair system and approaches the electron transport chain (ETC) system, which produces oxygen-free radicals [47]. Existing studies have shown that mtDNA mutations contribute to many diseases, such as PCOS.

## PCOS and OS

OS is the imbalance between oxidants and antioxidants that leads to the disruption of redox pathways and molecular damage [48]. It is the main pathophysiological mechanism of various human diseases [49], including T2DM, atherosclerosis, obesity, and IR, as well as biological aging [50–52]. Numerous investigations have shown that increases in OS and reductions in antioxidant concentration are associated with the characteristics of PCOS [53]. The first report showing that women with PCOS feature elevated OS was published two decades ago [54]. Subsequently, a growing number of studies have confirmed this phenomenon. For example, studies that compared the OS indices of women with PCOS and their age- and body mass index (BMI)-matched control counterparts found that women with PCOS have significantly higher GPx, GSH reductase activity, carbonyl, AOPPs, and MDA levels and lower TAC and TAOS levels [55–57]. The above results indicate that patients with PCOS have elevated OS and reduced antioxidant capacity. The study suggested that increased OS and reduced antioxidants are important for the PCOS phenotype, such as obesity, IR, inflammation, and hyperandrogenism. Obese patients have more severe OS levels, and significant correlations exist between OS markers and obesity indices (such as BMI and waist circumference) [58, 59]. Although the full mechanism of OS induced IR remains unclear, OS has been demonstrated to play crucial roles in IR pathogenesis. PCOS patients have an increased risk of developing metabolic syndrome, which may be related to OS [60]. OS is closely related to inflammation, and completely distinguishing inflammation from OS is difficult; they usually accompany each other [61]. OS contributes to hyperandrogenism in PCOS patients; in many studies, OS has been found to be positively correlated with androgen levels [62, 63]. In vitro, OS enhances the activity of ovarian steroid-producing enzymes and stimulates androgen production, whereas antioxidant chemicals, such as statins, inhibit their activity [64]. In another research, Hacer et al. found that women with PCOS have higher total oxidant status, oxidative stress index (OSI), and lower caspase 9 levels than the control group. Caspase 9 level is negatively related to total oxidant status. Caspase 9 is a member of the caspase family and is important during apoptosis [65]. Apoptosis is related to reduced follicular atresia, which is the mechanism underlying abnormal ovulation in PCOS. This study verified that the decrease in some apoptotic markers and increase in OS may interact in the progression of PCOS [66]. Comparing the OS statuses of follicular fluids from patients with PCOS and healthy women revealed that women with PCOS have increased MDA levels and lower TAC and thiol concentrations than healthy women. Follicular fluid contains protein, sugar, ROS, antioxidants, and hormones. The concentration of these substances directly affects oocyte maturity and quality. Imbalances between antioxidant factors and ROS in follicular fluid may have adverse effects on oocyte quality, fertilization, and embryo development [67] through altering the equilibrium of the follicular microenvironment [68] and result in abnormal ovulation and infertility in patients with PCOS [69]. Numerous indicators of OS are abnormal in the blood and follicular fluid of patients with PCOS. Therefore, the increase in OS plays an important role in the development of PCOS. It may be associated with the caspase system and lead to unusual apoptosis. However, its specific mechanism has not been investigated and should be clarified.

## PCOS and mitochondrial genome abnormalities

### Mitochondrial DNA copy number

Each mammalian cell contains approximately 1000–10,000 mtDNA copies. The transcription level of mtDNA principally depends on the number of mtDNA copies [70]. For example, tissues with high energy consumption, such as the heart, possess a high number of mtDNA copies. Mature oocytes usually contain at least 100,000 mtDNA copies, which are necessary for normal development [71]. The number of mtDNA remains stable, and the maintenance of mtDNA copy numbers at an appropriate level is crucial for maintaining mitochondrial function and cell growth [72]. Changes in mtDNA copy number reflect mitochondrial disorders caused by environmental oxidants and gene–environment interactions and are risk factors of several diseases. The change in mtDNA copy number is related to the occurrence and development of PCOS. For example, Lee and his partners found that women with PCOS have lower mtDNA copy numbers than those without PCOS. The mtDNA copy number of patients with PCOS is negatively correlated with IR level, waist circumference, and triglyceride levels and positively associated with SHBG levels. The decrease in SHBG not only increases the biological activity of androgen, but is also closely related to IR and metabolic syndrome [73]. This study suggests that alterations in mtDNA copy number may be associated with the development of PCOS, and the extent of mtDNA copy alteration is associated with the severity of PCOS [74]. The study suggested that alterations in mtDNA play a fundamental role in the increase in ROS [75], and decreased mtDNA content in peripheral leukocytes is associated with the development of T2DM [76, 77], which is the late-stage complication of PCOS. MtDNA copy number is also an indicator of fertilized potential and oocyte maturation. In mammals, an initial oocyte has 500 mtDNA copies, and the MII oocyte has 150,000–700,000 mtDNA copy numbers [78]. The number of mtDNA copies increases by more than 30 times during oocyte maturation [79]. The wide range of mtDNA copy number indicates a high degree of variability among individual human oocytes. The optimal mitochondrial copy numbers and adequate ATP levels (at least 2 pMol) are prerequisites for normal follicular development and maturation [80] to ensure the good developmental potential of blastocysts after fertilization [81]. The development potential of oocytes with low mtDNA copy numbers is significantly reduced [82], thereby reducing blastocyst formation [83]. This manifestation is consistent with the common symptoms of PCOS patients, such as anovulation and infertility. Several studies have obtained similar results [84, 85]. However, other studies have suggested that PCOS is related to the increase in mtDNA copy number, and this increase is regarded as a feedback response that compensates for mitochondrial dysfunction and respiratory chain damage or mtDNA mutation [37]. An induced pluripotent stem cell (iPSC) model was established from the somatic cells of patients with PCOS. iPSCs from women with PCOS present impaired mitochondrial respiration function and glycolytic ability and increased mitochondrial copy numbers and biosynthesis than non-PCOS patient-derived iPSCs. Furthermore, consistent with the increase in mtDNA copy numbers, the expression levels of mitochondrial biogenesis-related genes, such as peroxisome proliferator-activated receptor (PPAR) gamma-co-activator 1a (PGC-1α), TFAM, and nuclear respiratory factor 1 (NRF-1) in iPSCs from patients with PCOS are high. NRF-1 is an important regulator of the nucleus-encoded subunits of mitochondrial respiratory complexes [86]. PGC-1α is a member of the nuclear regulatory protein family and is involved in mitochondrial gene transcription regulation. PGC-1α can bind and coactivate the transcriptional function of NRF-1 on the TFAM promoter and regulate the replication and transcription of mtDNA [87]. Promoting glucose transporter (GLUT) is essential for intracellular glucose transport. Glucose restriction is reportedly associated with GLUT1 deficiency, which leads to decreased mitochondrial function, such as decreased MMP and activation of mitochondrial-dependent apoptosis. This study showed that the level of GLUT1 and GLUT3 decreased in PCOS patient-derived iPSCs. Therefore, decreased expression of GLUTs and related mitochondrial dysfunction may be associated with IR in PCOS. Mitochondrial biogenesis may be increased to compensate for the disruption of the functional integrity of mitochondria and the mitochondrial dysfunction of iPSCs from patients with PCOS [88]. In my opinion, these paradoxical results may be induced by different stages of disease manifestation. The abnormal metabolic state at the early stage of PCOS requires additional energy to stimulate the biogenesis of numerous mitochondria as a compensatory effect. This requirement increases mtDNA copy number. Mitochondria cannot sustain normal function with disease development, and the mtDNA copy number begins to decrease. This condition may be attributed to different samples. Pluripotent stem cells have strong development potential and metabolic rate. Thus, their mtDNA copy number may be higher than that of other cells. In conclusion, the abnormal number of mtDNA copies in patients with PCOS may play a role in the pathogenesis of PCOS.

### Mitochondrial gene mutations

The appearance of PCOS familial aggregation indicates that genetic factors play a significant role in its etiology [89]. Mitochondria are controlled by dual genes, and mutations in these genes may lead to respiratory chain dysfunction and subsequently result in reduced ATP production and excessive ROS production. Point mutations in the genes that encode mitochondrial transfer RNA (mt-tRNA) may be involved in the development of various diseases [90]. The mutations of mt-tRNA influence the structure and function of mitochondrial RNA. These influences include destabilizing the tertiary structure of mt-tRNAs, changing RNA precursor processing, nucleotide modification deletion, and insufficient aminoacylation [91]. Zhou et al. found six variants of mitochondrial tRNA genes, such as tRNA^Gln^, tRNA^Cys^, tRNA^Asp^, tRNA^Lys^, tRNA^Arg^, and tRNA^Glu^, and 7 variants in 12S ribosomal RNA (rRNA) gene, 3 variants in 16S rRNA in the peripheral blood of patients with PCOS. These mutations have emerged in highly conserved tRNA nucleotides that are crucial for the stability and biochemical function of tRNA [92]. These mutations have been found to be associated with T2DM and hypertension, which are long-term complications of PCOS patients. Thus, they inferred that these mutations may influence the balance of metabolism and participating in the occurrence of PCOS. Another study conducted by the same team identified the C3275T mutation in tRNA^Leu^, T4363C mutation in the anticodon stem of tRNA^Gln^, and A8343G mutation in tRNA^Lys^ in the mitochondrial genomes of patients with PCOS. Biochemical analysis has shown that the patients who displayed these mt-tRNA mutations have reduced MMP levels, ATP production, and mtDNA copy number but significantly higher ROS generation than those without the above mutations. Such tRNA mutations have been proven to be associated with metabolic abnormalities, such as hypomagnesemia, hypertension, and hypercholesterolemia. Detailed mechanisms are not yet clear, but these abnormalities are related to PCOS [93]. These results indicate that the mitochondrial dysfunctions caused by these mutations may be responsible for the clinical phenotypes of PCOS [84]. Few studies have explored the relationship between mt-tRNA mutations and PCOS. The clinical significance of mt-tRNA abnormalities cannot be clarified through these studies and should be confirmed through multiethnic and multicenter investigations. The mtDNA displacement loop (D-loop) is the only noncoding region of the mitochondrial genome. It plays an important role in mtDNA replication and transcription because it contains the starting point and promoter for the replication of the heavy and light chains of mtDNA [94]. Mutations in this region will result in mitochondrial dysfunction by altering mtDNA replication and transcription. The relationships between point mutations in the mtDNA D-loop and several human diseases has been reported [95–97]. A study sequenced the mitochondrial D-loop in 118 patients with PCOS and 114 South Indian controls and showed that a significant correlation exists between D310 and A189G single-nucleotide polymorphisms (SNPs) and PCOS. In addition, the number of mtDNA copies in patients with PCOS with D310 and A189G alleles is significantly lower than that in non-carriers. The changes in mtDNA D-loop and copy numbers may be a heritable risk factor for PCOS in South Indian women [85]. Several mtDNA D-loop variants and haplogroups have reportedly been associated with obesity in different ethnic populations [98]. For example, D-loop variants m.16292C > T and m.16189 T > C are associated with obesity in Germany [99]. Obesity is a common phenotype of PCOS; thus, the mutation of mtDNA D-loop may participate in the development of PCOS by affecting lipid metabolism.

Some researchers have indicated that mitochondrial biogenesis and mitochondrial ETC-related genes are abnormally expressed in patients with PCOS. Skov et al. found that the mitochondria are the most downregulated cellular component in the muscles of patients with PCOS. Electron transport from ubiquitin to cytochrome C is the most downregulated biological process. Nucleus-encoded OXPHOS-related genes (NDUFA3, SDHD, UCRC, COX7C, and ATP5H) decreased. These findings provide evidence showing that mitochondrial oxidative metabolism is impaired and that mitochondrial functional genes are downregulated in the skeletal muscle of women with PCOS [100]. The expression of NDUFA3 is significantly correlated with mtDNA copy number [101], which has a close relationship with follicular development and IR. The study suggests that SDHD SNPs are associated with BMI and obesity risk [102]. The knockdown of COX7C leads to the accumulation of myocardial fat tissue [103], and this gene is inactivated in obesity [104]. Another research has shown that the genotype distribution and allele frequency of the PGC-1α Gly482Ser polymorphism in patients with PCOS is significantly different from those of the PGC-1α Gly482Ser polymorphism in the matched group. The carriers of the PGC-1α rs8192678 “Ser” allele may have an increased possibility of developing PCOS [105]. The finding shows the significant association between the Gly482Ser variant of the PGC-1α gene and reduced insulin sensitivity in obese subjects; it also suggests the primary role of the PGC-1α gene on genetic susceptibility to IR in obesity [106]. Many studies on mitochondrial gene expression have proven that numerous alternations and mutations exist in patients with PCOS. Most of the abnormally expressed genes in these studies are involved in the pathogenesis of PCOS by affecting glycolipid metabolism. Genes are the internal factors that determine the normal operation of mitochondria. Their changes lead to the dysfunction of the executor protein and are succeeded by the occurrence of diseases. However, whether defects are directly related to the occurrence of PCOS requires further verification [Table 1].

**Table 1:** Mitochondrial Genome Abnormalities related to PCOS

GENE	Abnormality	References
mtDNA copy numbers	↑ / ↓	[74, 84, 85, 88, 107]
PGC-1α	↑/ methylated	[88, 108]
D-loop	methylated	[107]
TFAM	↑	[88]
NRF-1	↑	[88]
tRNA^Gln^	mutation	[92]
tRNA^Cys^	mutation	[92]
tRNA^Asp^	mutation	[92]
tRNA^Lys^	mutation	[92]
tRNA^Arg^	mutation	[92]
tRNA^Glu^	mutation	[92]
C3275T mutation in tRNA^Leu^	mutation	[84]
T4363C mutation in tRNA^Gln^	mutation	[84]
A8343G mutation in tRNA^Lys^	mutation	[84]
D310 in the mtDNA D-loop	SNPs	[85, 92]
A189G in the mtDNA D-loop	SNPs	[85]
NDUFA3	↓	[100]
SDHD	↓	[100]
UCRC	↓	[100]
COX7C	↓	[100]
ATP5H	↓	[100]
PGC-1α rs8192678 “Ser” allele	mutation	[100]
A3302G in mt-tRNA^Leu (UUR)^	mutation	[109]
C7492T in mt-tRNA^Ser (UCN)^	mutation	[109]
T12338C in ND5	mutation	[109]
NIX	↑	[108]
Rheb	↑	[108]

## Obesity and mitochondrial abnormalities

Approximately 50% of patients with PCOS are overweight or obese [110]. Obesity has a negative impact on female fertility and is associated with anovulation, miscarriage, and several pregnancy complications [111]. Abdominal obesity is related to IR, hyperandrogenism, chronic anovulation, and inflammation in patients with PCOS [112]. Mitochondria may be the main organ that leads to impaired energy metabolism in obese patients with PCOS. Increased OS can induce obesity by promoting preadipocyte proliferation and adipocyte differentiation, by increasing the size of mature adipocytes [113], and by stimulating hypothalamic neurons to reduce satiety and increase hunger behavior [114]. The efficiency of mitochondrial OXPHOS coupling in the skeletal muscle of obese (BMI > 33 kg/m^2^) women is reduced, and the mitochondrial H_2_O_2_ emissions of obese women are higher than those of lean (BMI < 23 kg/m^2^) women. These conditions reflect the impaired mitochondrial bioenergetics and increased OS exhibited by obese women [115, 116]. Under obesity conditions, the increased accumulation of lipid and fatty acids in ovaries, oocytes, and surrounding cumulus cells disturbs oocyte metabolism, hinders mitochondrial function, and damages oocyte quality by increasing mitochondrial damage [117]. Thus, obese women with PCOS frequently wait longer for a successful pregnancy and have a higher risk of miscarriage than non-obese women [118]. Oocytes from high-fat diet-exposed obese mice have abnormal morphologies, as well as lower levels of ATP, citrate, and creatine phosphate and higher levels of ROS when compared with oocytes from mice on a normal diet. Similar research has shown that maternal diet-induced obesity in mice can intensify ROS generation and GSH depletion. These results are indicative of increased OS in the oocytes and zygotes of obese mice. Moreover, mitochondria are evenly distributed throughout the ooplasm of eggs from lean females but are discontinuously distributed in the ooplasm of eggs from obese females, and high-density clusters are concentrated around the cortical ooplasm and nucleus [117]. The normal structure of mitochondria in the cells is important for signaling events relevant to fertilization. Thus, obese women, such as obese patients with PCOS, are vulnerable to anovulation and infertility [119].

Mitochondrial energetics is mainly controlled by the coordination between supply and demand. Thus, energy restriction can reduce the production of oxidants, increase the scavenging ability of antioxidants, and reduce oxidative damage to DNA and protein [120]. Weight loss through vertical banded gastroplasty is accompanied by remarkable reductions in the production of the free radical MDA. Surgical weight loss can remarkably reduce the generation of oxidants and increase the levels of vitamins E, A, and beta-carotene, which play a key role in non-enzymatic antioxidant defense in biological systems [121]. Dandona et al. found significant reductions in ROS production from leucocytes and the oxidative damage of lipids, proteins, and amino acids in obese people after a short period of weight loss through dietary restriction [122]. Weight loss may reverse mitochondrial dysfunction. Obesity is a common manifestation of PCOS and is closely related to mitochondrial dysfunction and elevated OS, which may accelerate the progress of PCOS in obese patients and aggravate metabolic disorders, such as IR, hyperandrogenism, and abnormal follicular development. Weight loss is the first treatment recommended for obese patients with PCOS. The drastic improvement in PCOS symptoms after weight loss may partly stem from the reduction in ROS production and the elevation of antioxidant levels.

## IR, Hyperandrogenism, and mitochondrial abnormalities

PCOS frequently occurs with IR and hyperandrogenism. IR is defined as the relative impairment of insulin effects on glucose, protein, and lipid metabolism in target tissues. IR is an important metabolic disorder associated with PCOS [123]. It is a widely recognized risk factor that affects the reproductive and metabolic functions of patients with PCOS. It can increase androgen levels and increase the risk of metabolic disorders, cardiovascular diseases, and tumors [124]. Hyperandrogenism is a diagnostic criterion of PCOS. Women with functional ovarian hyperandrogenism feature considerably elevated basal insulin secretion rates and weakened dietary stimulate insulin [125]. IR and hyperandrogenism are closely related, and their occurrence are indispensable for mitochondrial dysfunction. Insulin is the main regulator of OXPHOS, and its secretion can directly affect mitochondrial function [126]. Conversely, mitochondria play a vital role in the normal function of insulin. The reduced effectiveness of insulin stems from increased ROS, which can induce the abnormal activation of serine/threonine kinase signaling pathways, such as c-Jun N-terminal kinase (JNK), nuclear factor kappa-B (NF-kB), and p38-mitogen-activated protein kinase (MAPK), and increase the phosphorylation of insulin receptors and insulin receptor substrate (IRS) proteins [127]. Accordingly, mitochondrial insufficiency could be the main reason for IR and PCOS-related complications [128]. A study divided 101 PCOS subjects into PCOS non-IR (Homeostasis model assessment, HOMA-IR < 2.5) and PCOS IR (HOMA-IR > 2.5) groups. The mitochondrial ROS production and myeloperoxidase (MPO) levels in patients with PCOS, especially those in patients with PCOS and IR, are elevated [129]. MPO is a hemoglobin that is released by activated white blood cells in inflammatory sites and can produce ROS [130]. Sabuncu et al. reported that MDA production in patients with PCOS is significantly higher than that in controls. MDA level is negatively correlated with insulin sensitivity. Moreover, the GSH level of patients with PCOS is significantly lower than that of the control group. The reduction in GSH level is associated with IR, which can reduce the level of GSH by inhibiting the activation of the GSH reductase system [131]. High MDA and low GSH are associated with impaired insulin sensitivity in patients with PCOS [54]. Rats in the PCOS-IR group also display higher MDA levels and lower TAC, SOD, and GSH levels than those in the control group [132].

The mutations of several genes related to mitochondrial OXPHOS have been identified and have been proven to participate in the progression of IR in patients with PCOS. A study revealed the existence of homologous A3302G in the receptor arm of the transfer RNA^Leu (UUR)^ gene that destroys a highly conserved base pairing (2 T-71A), a homologous mutation C7492T in the mt-tRNA^Ser (UCN)^ gene, and ND5 T12338C mutation in women with PCOS. The effects of these mutations on mt-tRNA metabolism may result in the inefficiency of mitochondrial protein synthesis and may be the cause of mitochondrial dysfunction among patients with PCOS and IR [109]. Skov et al. discovered that the expression levels of OXPHOS-related genes (NDUFA3, SDHD, UCRC, COX7C, ATP5H, and PGC-1ɑ) and ribosomal proteins in skeletal muscle biopsies collected from women with PCOS and IR are unanimously decreased [100]. Previous studies have found a similar reduction in the expression of these genes in patients with T2DM and their first-degree relatives [133–135]. The influence of pioglitazone on the expression of mitochondrial-related genes in the skeletal muscle of patients with PCOS with IR has been investigated. Pioglitazone is a PPAR-γ agonist that can induce adipogenesis and insulin sensitization and has anti-diabetes effects [136]. It drastically upregulates the expression of genes related to OXPHOS and ribosomal protein biosynthesis in obese patients with PCOS and corrects gene downregulation. These findings confirm the hypothesis that the alteration of mitochondrial gene expression may be a key factor in the pathological characteristics of IR with PCOS and may increase the risk of patients with PCOS to develop T2DM [137]. Pancreatic beta cell dysfunction may be either an important pathogenic determinant of IR in PCOS [138]. P47^phox^ (phox, phagocytic oxidase) is a key protein component of nicotinamide adenine dinucleotide phosphate (NADPH) oxidase, and its expression is negatively correlated with insulin sensitivity and beta cell function. Glucose intake further stimulates ROS production by nicotinamide adenine dinucleotide phosphate oxidase by enhancing the translocation of p47^phox^ within the membranes of circulating monocytes [139]. Women with PCOS exhibit a higher levels of ROS generation and p47^phox^ protein accompanied with higher glucose-stimulated insulin secretion and a tendency for beta cell function failure than controls [62, 140]. OS significantly decreases with the improvement of insulin sensitivity. By contrast, OS reduction through the inhibition of NADPH oxidases can reverse IR in animal models [113]. This condition completely illustrates the relationship between IR and increased OS and provides a novel perspective for the treatment of IR in patients with PCOS.

Hyperandrogenism and abnormal mitochondrial function interact with each other. Androgen overexposure causes IR in female mice by inducing OS elevation and pancreatic beta cell failure and may account for the high occurrence of IR in patients with PCOS. A study compared glucose-induced insulin secretion and mitochondrial function in the islets of dihydrotestosterone (DHT)-induced and control rats and found that oxygen consumption rate, ATP production, and mitochondrial copy number reduced in DHT-induced rats. The number of mtDNA copies showed the most drastic reduction and decreased by approximately 40%. The expression levels of several key genes, including TFAM, PGC-α, and NRF1, and mtDNA-encoded genes, such as nicotinamide adenine dinucleotide (NADH) dehydrogenase subunit 1, NADH dehydrogenase subunit 3, mitochondrial respiratory chain complex II, mitochondrial respiratory chain complex IV, and ATP synthase subunit b in DHT-treated rat islets reduced [141]. Glucose-stimulated insulin secretion drastically dropped. Androgens can induce mitochondrial function disorder in vitro and cause beta cell failure in an androgen receptor-dependent manner [142]. OS does not increase and beta cell function is not impaired when androgen receptors are blocked. Liu et al. used control female mice (CF) and androgen-resistant testicular feminization (TFM) mice. Beta cell stress in TFM mice was induced through streptozotocin (STZ) treatment. They found that CF mice with STZ-induced beta cell stress are susceptible to beta cell failure and insulin-deficient T2DM after treatment with testosterone or DHT. By contrast, testosterone treatment does not induce beta cell failure in TFM mice. Testosterone or DHT treatment provokes systemic OS in CF mice but eliminates systemic OS in TFM mice. Therefore, the over-activation of androgen receptors by testosterone may result in systemic OS. In the case of pre-existing beta cell stress, systemic OS may lead to beta cell failure and IR [143]. IR and hyperandrogenism are mutually enhanced in patients with PCOS. Studies have proven that abnormal mitochondrial function is involved in the occurrence and development of IR and hyperandrogenism. However, the relationship between the two deficits under the mediation of the mitochondrial pathway should be investigated.

## Abnormal follicular development and mitochondrial abnormalities

PCOS accounts for more than 75% of cases of anovulatory infertility, which is frequently succeeded by impaired follicular maturation, anovulation, and biochemical pregnancy. Oocytes have a large quantity of mitochondria that play an important regulatory role in oocyte maturation [144], fertilization, and pre-implantation embryo development [145]. At the beginning of each month, a group of oocytes begins to grow and develop together. Only one oocyte, however, completes meiosis I. This oocyte is the dominant oocyte. This process is accompanied by an elevation in ROS levels and the inhibition of antioxidant production. Conversely, the successful division of meiosis II requires the protection of antioxidants, such as CAT, SOD, GSH, transferase, and paraoxanase [146]. This requirement indicates that the interaction between ROS and antioxidants in the ovaries is complex. ROS in follicles mainly originate from macrophages, neutrophils, and granulocytes. Follicular ROS promotes apoptosis in most follicles, and GSH and Follicle stimulating hormone (FSH) play the main role in counterbalancing follicle apoptosis. A study has demonstrated that inhibiting GSH synthesis can increase the rate of antral follicular atresia in rats [147]. The drastic increase in MMP during oocyte maturation [148] is related to the increased rate of OXPHOS [149]. The reduction in MMP is related to damage to oocyte development potential [150, 151]. Changes in the levels of tricarboxylic acid pathway (TCA) cycle and nicotinamide adenine dinucleotide (NAD) catabolism in the follicular fluid of patients with PCOS are accompanied by disordered redox potential and increased OS in cumulus cells (CCs). Moreover, mitochondrial biogenesis rate, mitochondrial DNA content, and MMP in the CCs of patients with PCOS are significantly lower than those in the CCs of the control group. In addition, in the PCOS group, the gene expression of PGC-1ɑ is downregulated, and the methylation rate of PGC-1ɑ promoter is high. DNA methylation is a characteristic and stable epigenetic modification. It mainly affects gene expression by destroying transcription factor binding and methyl binding protein attraction and initiating chromatin compression and gene silencing [152]. Therefore, the methylation of the PGC-1a promoter will reduce the mitochondrial biogenesis rate by inhibiting the transcription of PGC-1a. Autophagy that occurs in mitochondria is called mitophagy. The expression levels of autophagy genes ATG9 and ATG12 and mitochondrial mitophagy receptors NIX and Rheb are considerably higher in patients with classic PCOS than those in the control group. The increase in ROS levels in GCs may induce GCs mitophagy. The imbalance of redox potential in follicular fluid and high OS, accompanied by the increased mitophagy of CCs and the methylation of several important genes in CCs, may be the potential pathological mechanisms of anovulation in PCOS [108]. Jia et al. obtained similar findings. They isolated gilt oocytes from normal and polycystic ovaries and assessed the quality of mitochondria and the methylation of related genes in oocytes. They found that the polar bodies of polycystic ovary (PCO) oocytes are extruded, and the structure of mitochondria are distorted. Moreover, MMP, and mtDNA copy numbers, as well as the expression of mtDNA coding genes in PCO oocytes, are drastically reduced. The mtDNA sequence encoding the 12S, 16S rRNA, and the D-loop region is methylated. Furthermore, cleavage and blastocyst rates reduced in PCO oocytes. These phenomena suggest that the hypermethylation of mtDNA and reductions in related gene expression may affect the mitochondrial function of gilts to a large extent and result in the decline of PCO oocyte quality [107]. Accordingly, Lai et al. found that the levels of ROS in the CCs of patients with PCOS are significantly higher and the success rate of in vitro fertilization-embryo transfer is lower than those in the control group [153]. Patients with PCOS display abnormal mitochondrial function, structure, and gene expression in their follicles. These conditions affect the development of follicles, ovulation, and fertilization. However, the detailed mechanism of these manifestations has not been investigated. Whether lipid, androgen, IR or other defects play a role and their interaction remain unclear.

## Inflammation and mitochondrial abnormalities

Inflammation participates in the pathogenesis of numerous chronic diseases. A relationship exists among chronic low-grade inflammation and the pathogenesis and development of PCOS [154]. This relationship is confirmed by the increase in the levels of circulatory markers associated with inflammation, such as C-reactive protein (CRP), IL-6, TNF-ɑ, interleukin-8 (IL-8), monocyte chemoattractant protein-1(MCP-1), soluble intercellular adhesion molecule-1, and white blood count (WBC) in patients with PCOS [22, 155, 156]. OS and chronic inflammation are two closely interconnected mechanisms. The release of numerous active substances by inflammatory cells in inflammatory sites results in the overgeneration of OS [157]. The initiation of intracellular signaling cascades by high levels of ROS can enhance the expression of proinflammatory genes [158]. Pandey et al. induced PCOS in rats through letrozole treatment. The levels of CRP and OS markers (lipid peroxidation, CAT, and SOD) in rats treated with letrozole have been significantly altered relative to those in the control group [154]. Similar results have also been found in humans. Isık et al. found that patients with PCOS have higher levels of mean WBC and mean CRP than controls. Xanthine oxidase (XO) activity increases in women with PCOS. XO is the source of ROS and can generate superoxide anion radicals. By contrast, SOD activity reduces in patients with PCOS. The mean CRP is positively correlated with XO activity and is negatively correlated with SOD activity in women with PCOS [159]. This condition suggests that changes in the OS index are closely related to the changes in inflammatory markers during the development of PCOS. The relationship between OS indices and inflammatory cytokines in the follicular fluid of patients with PCOS and women with normal ovarian function was investigated recently. Patients with PCOS have higher levels of MDA and TOS and lower levels of TAC and thiol than the controls. Patients with PCOS have higher levels of IL-6, IL-8, and TNF-ɑ and lower concentrations of interleukin-10 (IL-10) than the controls. IL-10 is an anti-inflammatory cytokine and inhibitor that can inhibit the expression of proinflammatory cytokines in inflammatory-related cells. Significant correlations have been found between MDA and TAOS concentrations with TNF-ɑ and between IL-6 and MDA, between IL-8 and TAC, between IL-10 and TAOS, and between IL-10 and TAC levels in the follicular fluid of patients with PCOS. TAC and thiol levels are negatively correlated with TNF-ɑ. The association between inflammation and potential interaction with increased OS in the blood and follicular fluid of women with PCOS may cause defects in oocyte development [160].

**Fig. 1:**
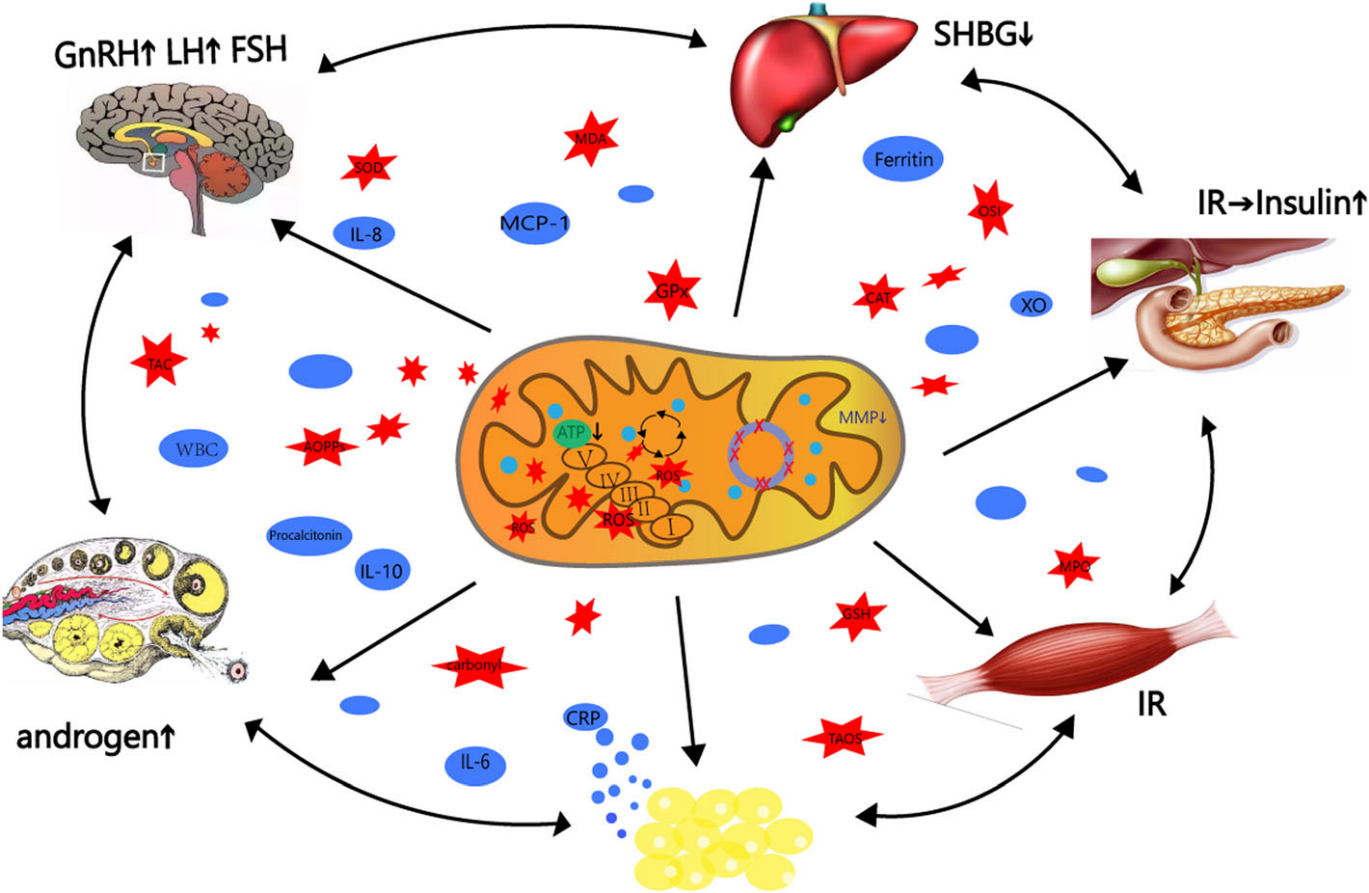
Mitochondrial dysfunction participates in multiple organs disorder, which are all involved in polycystic ovary syndrome physiopathology are described briefly in the figure. Polycystic ovary syndrome is caused by a vicious cycle of androgen excess, insulin resistance, low-grade inflammation, obesity and increased oxidative stress

A previous study has suggested that TNF-ɑ can promote androgen production in vitro by stimulating the proliferation of theca cells and intensifying the effect of insulin in a dose-dependent manner [161]. By contrast, androgen can indirectly participate in the occurrence of low-grade inflammation by acting on adipose tissues. Androgen can stimulate adipocyte hypertrophy and induce the secretion of inflammatory factors [162]. Zhang showed that hyperandrogenism can promote increases in IL-6, MCP-1 mRNAs, and TNF-ɑ protein levels. Therefore, hyperandrogenism may be essential and can aggravate abnormal inflammation in patients with PCOS [163]. Inflammation might be associated with other prominent aspects of PCOS, such as IR and obesity. The levels of IL-6, TNF-α, and adhesion molecules (vascular cell adhesion molecule 1, VCAM-1 and E-selectin) are increased in women with PCOS with IR. The mitochondrial dysfunction that accompanies the increments in IL-6, TNF-α, VCAM-1, and E-selectin is indicative of reduced mitochondrial O_2_ consumption and GSH and MMP levels and elevated ROS production [164]. TNF-ɑ can weaken insulin signaling by reducing the activity of insulin receptor tyrosine kinase and impairing the development of insulin receptors; these effects favor the development of IR [165] and suggest that mitochondrial dysfunction and inflammation are involved in the occurrence of IR in patients with PCOS. Adipose is an endocrine organ that produces numerous adipokines, such as leptin, TNF-ɑ, IL-6, and adiponectin, which can directly lead to inflammation-associated metabolic abnormalities [166]. Obesity-associated inflammation is initially triggered by redundant nutrients that initiate the activation of several metabolic signaling pathways, such as the JNK, NF-kB, and protein kinase R pathways [167, 168]. The activation of these pathways leads to the production of several inflammatory cytokines and results in chronic low-grade inflammation [169]. Rashad et al. suggested that overweight and obese women with PCOS have significantly higher serum procalcitonin, CRP, WBC, and neutrophil counts than lean subjects [170]. Procalcitonin is a marker of low-grade inflammation that is released into the blood as a response to systemic inflammation [171]. Ferritin is also another marker of low-grade inflammation, and obese patients with PCOS have high ferritin levels [172, 173]. Weight loss drastically reduces the levels of inflammation markers in obese patients [174]. The interactions of chronic low-grade inflammation with increased OS are prevalent in patients with PCOS and are closely related to other manifestations of PCOS, such as IR, hyperandrogenism, and obesity, which influence and promote each other. This condition confirms the complexity of the pathogenesis of PCOS. Inflammation is one of the body’s defense mechanism against lesions. It is only a participant, not a direct pathogenic factor, in the development of PCOS. Whether inflammation alone can induce PCOS in humans should be investigated [Fig. 1].

## OS as a target of PCOS therapies

No consensus on the treatment of PCOS exists because the etiology of this disease remains unclear. Therapies for PCOS are usually selected on the basis of clinical manifestations and expected effect. At present, treatment mainly aims to alleviate PCOS symptoms by inhibiting excessive androgen secretion, improving menstrual dysfunction, protecting the endometrium, promoting fertility, and alleviating metabolic disorders [175]. The exact mechanisms of OS that affect the pathogenesis of PCOS remain unclear. OS, however, is related to the progression of PCOS. A large number of studies involving in vitro and in vivo animal experiments have proven that antioxidants play a key role in reducing OS levels. Therefore, alleviating PCOS by inhibiting OS provides a new perspective for the treatment of PCOS.

Women with PCOS receiving vitamin D and omega-3 fatty acid co-supplementation have significantly reduced serum MDA levels and elevated plasma TAC levels [176]. A meta-analysis of randomized controlled trials has shown that women with PCOS taking vitamin D supplements have significantly decreased MDA and CRP levels and increased TAC levels [177]. Coenzyme Q10 (CoQ10) is an antioxidant component of the ETC. CoQ10 supplementation can improve mitochondrial distribution, spindle formation, and chromosome alignment in the oocytes of obese mice [116]. SOD and GSH levels increased and MDA levels decreased in PCOS rats with IR after 8 weeks of CoQ10 treatment [132]. Metformin intake could reduce glucose and FSH, increase mitochondrial O_2_ consumption, MMP, mitochondrial mass, and GSH levels, and decrease ROS and H_2_O_2_ production in subjects with PCOS. Metformin is clinically used to improve IR in patients with PCOS, and its capacity to reduce ROS has only been recently discovered [178]. Selenium supplementation may protect against OS and inflammation by attenuating the formation of ROS and regulating cell signaling pathways [179]. Probiotics can improve antioxidant capability and hormonal disorders by alleviating IR and exerting anti-inflammatory effects [180, 181]. Probiotics and selenium co-administered for 12 weeks to women with PCOS significantly reduced MDA levels and significantly increased TAC and total GSH levels compared with the placebo [182]. Women with PCOS showed a significant reduction in serum MDA levels after treatment with atorvastatin. Reductions in MDA concentrations are correlated with reductions in CRP and total testosterone [183]. These approaches are novel attempts to reduce OS in patients with PCOS and ameliorate symptoms. Nevertheless, their clinical application remains to be verified. Specific mechanism-based strategies that target OS pathways to improve the outcome of PCOS exist and represent considerable progress in the treatment of PCOS. Antioxidant therapy alone cannot prevent PCOS, which is a complex disease. However, a combination of drugs may drastically increase the efficiency of treatment. The next step is to find the most appropriate PCOS treatment through clinical trials.

## Conclusion

The above review supports the hypothesis that abnormal mitochondrial metabolic markers and related genes are associated with and participate in the occurrence of PCOS. Not all aspects of evidence are covered in depth in this review because of space limitations. In conclusion, patients with PCOS exhibit several mitochondrial gene mutations, deletions, and nucleotide variations and mitochondrial dysfunction indexes. The effects of these changes on the progression of PCOS require verification. For example, the abnormal mitochondrial-related gene in the animal is knocked down, and whether or not the animals exhibited PCOS phenotypes is observed. If they show this phenotype, then the mitochondrial genetic abnormalities cause PCOS. On the contrary, drug or physical stimulation can be used to increase the level of OS in animals. After a period of time, we can also observe whether they show abnormal ovulation, IR, and other phenotypes to determine whether abnormal mitochondrial function can induce the onset of PCOS. Like this, the etiopathogenesis of this disease with mitochondrial dysfunction should be investigated, and various effective treatments should be developed.

## Data Availability

This review was based on published data.

## Abbreviations

·OH: hydroxyl radicals
1O2: Singlet oxygen
AOPPs: Advanced oxidation protein products
ATP: Adenosine triphosphate
BMI: Body mass index
CAT: Catalase
CCs: Cumulus cells
CF: Control female
CoQ10: Coenzyme Q10
CRP: C-reactive protein
DHT: Dihydrotestosterone
D-loop: Displacement loop
ETC: Electron transport chain
FSH: Follicle stimulating hormone
GLUT: Promoting glucose transporter
GPx: Glutathione peroxidase
GSH: Glutathione
H_2_O: water
H_2_O_2_: Hydrogen peroxide
HOMA: Homeostasis model assessment
IL-10: Interleukin-10
IL-6: Interleukin-6
IL-8: Interleukin-8
iPSC: induced pluripotent stem cell
IR: Insulin resistance
IRS: Insulin receptor substrate
JNK: c-Jun N-terminal kinase
LH: Luteinizing hormone
MAPK: Mitogen-activated protein kinase
MCP-1: Monocyte chemoattractant protein-1
MDA: Malondialdehyde
MMP: Mitochondrial membrane potential
MPO: Myeloperoxidase
mtDNA: mitochondrial genome
mt-tRNA: mitochondrial transfer RNA
NAD: Nicotinamide adenine dinucleotide
NADH: Nicotinamide adenine dinucleotide
NADPH: Nicotinamide adenine dinucleotide phosphate
NF-kB: Nuclear factor kappa-B
NRF-1: Nuclear respiratory factor 1
O2·−: superoxide anions
OS: Oxidative stress
OSI: Oxidative stress index
OXPHOS: Oxidative phosphorylation
PCOS: Polycystic ovary syndrome
PGC-1α: Peroxisome proliferator-activated receptor gamma-co-activator 1a
Phox: Phagocytic oxidase
PPAR: Peroxisome proliferator-activated receptor
ROS: Reactive oxygen species
rRNA: ribosomal RNA
SHBG: Sex hormone binding protein
SNPs: Single-nucleotide polymorphisms
SOD: Superoxide dismutase
STZ: Streptozotocin
T2DM: Type 2 diabetes
TAC: Total antioxidant capability
TAOS: Total antioxidant status
TCA: Tricarboxylic acid pathway
TFAM: Transcription factor A
TFM: Testicular feminization mice
TNF-^α^: Tumor necrosis factor-^α^
VCAM-1: Vascular cell adhesion molecule-1
WBC: White blood count
XO: Xanthine oxidase
